# Physico-chemical and microbiological characterization of spontaneous fermentation of Cellina di Nardò and Leccino table olives

**DOI:** 10.3389/fmicb.2014.00570

**Published:** 2014-10-28

**Authors:** Gianluca Bleve, Maria Tufariello, Miriana Durante, Ezio Perbellini, Francesca A. Ramires, Francesco Grieco, Maria S. Cappello, Stefania De Domenico, Giovanni Mita, Maria Tasioula-Margari, Antonio F. Logrieco

**Affiliations:** ^1^Unità Operativa di Lecce, Consiglio Nazionale delle Ricerche - Istituto di Scienze delle Produzioni AlimentariLecce, Italy; ^2^Agricola Nuova Generazione Soc. Coop.Martano, Italy; ^3^Department of Chemistry, Section of Food Chemistry, University of IoanninaIoannina, Greece; ^4^Consiglio Nazionale delle Ricerche- Istituto di Scienze delle Produzioni AlimentariBari, Italy

**Keywords:** table olives, yeast, lactic acid bacteria, volatile compounds, fermented food

## Abstract

Table olives are one of the most important traditional fermented vegetables in Europe and their world consumption is constantly increasing. In the Greek style, table olives are obtained by spontaneous fermentations, without any chemical debittering treatment. Evolution of sugars, organic acids, alcohols, mono, and polyphenol compounds and volatile compounds associated with the fermentative metabolism of yeasts and bacteria throughout the natural fermentation process of the two Italian olive cultivars Cellina di Nardò and Leccino were determined. A protocol was developed and applied aimed at the technological characterization of lactic acid bacteria (LAB) and yeast strains as possible candidate autochthonous starters for table olive fermentation from Cellina di Nardò and Leccino cultivars. The study of the main physic-chemical parameters and volatile compounds during fermentation helped to determine chemical descriptors that may be suitable for monitoring olive fermentation. In both the analyzed table olive cultivars, aldehydes proved to be closely related to the first stage of fermentation (30 days), while higher alcohols (2-methyl-1-propanol; 3-methyl-1-butanol), styrene, and o-cymene were associated with the middle stage of fermentation (90 days) and acetate esters with the final step of olive fermentation (180 days).

## Introduction

Olive production is considered one of the major agronomic practices of Mediterranean countries such as Italy, Greece and Spain that together supply almost 30% of world olive annual production (IOC, 2014). Table olives are one of the most important traditional fermented vegetables in Southern European countries and their consumption is constantly increasing. Italy produces about 76,000 tons/year of table olives, which is 3.1% of world and 10.3% of European Union production, respectively. Leccino, a cultivar originary from Tuscany and Umbria regions (Central Italy), is the most widespread dual-purpose olive variety in the world, due to its exceptional adaptability to different growing conditions (Vossen, 2007). Cellina di Nardò is an olive cultivar autochthonous of Salento (Apulia, Southern Italy). The production of this traditional cultivar is about 180,000 tons per year. It is mostly used to produce olive oil and in part it is used for the production of table olives that are being highly appreciated and requested outside the Salento area. Natural black olives in brine (Greek-style), Spanish-style green olives and black oxidized olives (also known as Californian-style) are the three most important commercial preparations of table olives on the international market (Garrido-Fernández, 1997). In the Greek-style production system, the fruits are placed directly into brine with a salt concentration of about 6–10% (w/v), thus allowing spontaneous fermentation to take place (Balatsouras, 1990). Spontaneous fermentation lasts 8–12 months and is driven by mixed populations of microorganisms, mainly the epiphytic microbial populations of yeasts and lactic acid bacteria (LAB) (Garrido-Fernández, 1997; Brenes et al., 2004; Romero et al., 2004; Hurtado et al., 2008). Lactic acid fermentation by LAB is considered the key step in spontaneous fermentation processes particularly in those related to directly brined olives (green or black), since it promotes: (i) debittering of the olives through oleuropein hydrolysis, (ii) lowering of brine pH, which prevents any growth of spoilage and pathogenic microorganisms (Spyropoulou et al., 2001; Caggia et al., 2004; Cawthorne et al., 2005), (iii) the enhancement of a correct flavor and texture profile in the final product (Ciafardini et al., 1994; Garrido-Fernández, 1997; Sanchez et al., 2000). However, the important role of yeasts in the table olive production process has recently been considered (Bevilacqua et al., 2013; Heperkan, 2013). The beneficial roles of yeasts consist of: (i) the production of volatile compounds and metabolites able to improve the organoleptic characteristics of the final product (Garrido Fernández et al., 1995), (ii) the release of nutritive compounds that enhance LAB growth (Arroyo-López et al., 2008; Nisiotou et al., 2010; Bautista-Gallego et al., 2011), (iii) activity against undesired microorganisms (Psani and Kotzekidou, 2006), and bioreduction of phenolic compounds (Ettayebi et al., 2003). On the other hand, yeasts can cause several problems during table olive production, such as the formation of gas pockets, the softening of olive tissue, package bulging, clouding of brines, and production of off-flavors (Turantas et al., 1999). At present, industrial production of black olives and several cultivars of green olives in local industries is carried out by spontaneous fermentation processes which are not predictable and are strongly influenced by the autochthonous microbiota, the physico-chemical conditions, the availability of fermentable substrates and salt content (De Castro et al., 2002; Tassou et al., 2002; Alvarez et al., 2003; Abriouel et al., 2011). Among the possible available technological approaches for controlling fermentation process, the use of *Lactobacillus plantarum* and *Lactobacillus pentosus* as starter cultures has been proposed (De Castro et al., 2002; Leal-Sánchez et al., 2003; Panagou et al., 2003, 2008; Marsilio et al., 2005; Servili et al., 2006; Sabatini and Marsilio, 2008), in order to avoid the unpredictability of spontaneous fermentations and to improve the production process. However, in recent years, the importance and the potential applications of yeasts as starters for table olive processing has been also recognized Arroyo-López et al., 2008, 2012b; Bevilacqua et al., 2013; Bonatsou et al., 2015.

The main aims of the present study were: (i) to characterize microbial population associated to Cellina di Nardò and Leccino table olive fermentations by technological and molecular approaches; (ii) to investigate the evolution of the main physico-chemical parameters and volatiles during fermentations for identifying specific molecules to be used as tools for fermentation monitoring; (iii) to correlate the outcome of physic-chemical analyses with the microbial growth.

## Materials and methods

### Olive samples and fermentation procedures

The lab-scale fermentations were performed in triplicate on olive samples of Cellina di Nardò and Leccino cultivars at an industrial plant belonging to Agricola Nuova Generazione (Martano, Lecce, Italy). Healthy black olives (90 kg) were collected at the black stage of ripening and washed with tap water to eliminate plant materials (residues of leaves, branches) and superficial contaminants. The olives were then selected (caliber above 10–12 mm), washed and placed in plastic vessels of 30 kg capacity filled with 20 L of 13% NaCl (wt/vol). The olives were allowed to ferment at ambient temperature, adopting, when required, correction of salinity by addition of salt.

### Isolation of microbial population

To isolate epiphytic yeasts, *Enterobacteriaceae* and LAB, a sample consisting of 15 olives for Leccino (32,41 g) and 20 olives of Cellina di Nardò (32,62 g) and 50 ml of brine was stirred on a rotary shaker at 200 rpm for 30 min. The sediment was recovered after centrifugation at 5000x g for 10 min at room temperature, and suspended in 0.5 ml of 0.1% (wt/vol) peptone water.

Salinity, pH, temperature and the formation of mold layers on top of the brine were evaluated during fermentation, at the following time points: 0, 5, 10, 15, 30, 45, 60, 75, 90, 105, 120, 135, 150, 165, 180 days. At each different fermentation time, olives and brines were collected, incubated together for 30 min in a rotary shaker (200 rpm) and then aliquots of brines were collected, diluted with one volume of sterile 100% glycerol and stored at −80°C for further analysis. In order to quantify LAB, *Enterobacteriaceae* and yeasts present in olives and brines, samples were serially diluted with 0.1% (wt/vol) peptone water and applied to agar plates containing the following media: Man, Rogosa and Sharpe Agar (MRS), added with 0.05 g/L of nystatin (incubation at 30°C under anaerobic conditions for 48–72 h); Violet Red Bile Glucose Agar (VRBGA; incubation at 37°C for 18–24 h); Sabouraud Dextrose Agar, added with 0.1 g/of ampicillin and 0.05 g/L of kanamycin (incubation at 25°C for 2–4 days). The population on each agar plate was then subjected to microbial count in order to quantify the LAB, *Enterobacteriaceae* and yeast concentrations in each sample. Thirty-five colonies were randomly selected from the agar plates (specific for LAB and yeasts) at the sampling times indicated above.

### Yeasts and LAB technological characterization

The set up of a protocol for the technological characterization of autochthonous starters for table olive production preliminarily required the formulation of a synthetic “model brine,” in order to use it as reliable substrate for the *in vitro* technological assays. For this purpose, two different synthetic brines were formulated, for yeast and LAB selection, respectively. For yeast selection, brine samples deriving from each olive cultivar were collected at days 15, 30, and 60 of fermentation and analyzed, being this period representative of selective conditions introduced by olives and brine after the yeasts started to increase their number. As described by Bleve et al. (2015), the following phenolic compounds were detected and quantified in the following ranges: tyrosol (11.39–256.18 mg/L), pyrocatechol (14.69–18.14 mg/L), caffeic acid (7.54–62.11 mg/L), oleuropein (26.30–900.04 mg/L), verbascoside (7.95–422.21 mg/L). The results helped to formulate the composition of the model brine as follows: 100 mg/L tyrosol, 30 mg/L caffeic acid, 500 mg/L oleuropein, 200 mg/L verbascoside, 10% NaCl, 3 g/L glucose, 0.5 g/L yeast extract, 20 g/L agar, pH 4–4.5. Microbial isolates were grown at 15°C. For LAB selection, brine samples were collected every 2 weeks starting from day 120 until day 180. Samples were analyzed to determine the qualitative and quantitative profile of phenolic compounds by reversed-phase HPLC-DAD, as described above. The main phenolic compounds present in several brines of table olive fermentations were identified and quantified at the specific time point when the LAB appeared: tyrosol (13.68–200.42 mg/L), caffeic acid (0–32.77 mg/L), oleuropein (51.58–667.33 mg/L), verbascoside (32.82–190.47 mg/L). The concentration of each phenolic compound detected was then considered to formulate model brines: 100 mg/L tyrosol, 15 mg/L caffeic acid, 300 mg/L oleuropein, 100 mg/L verbascoside, 8% NaCl, 3 g/L glucose, 0.5 g/L yeast extract, 20 g/L agar, pH 4.2. Bacterial isolates for each of the two olive cultivars were applied on MB and incubated for 15 days at 15°C. Beta-glucosidase activity was determined by replica plating the yeast onto selective media, for yeast [SC = 0.67% yeast nitrogen base (YNB), 0.5% arbutin, 2% agar, pH 5.0] and for bacteria (MRS medium, 0.5% arbutin, 2% agar, pH 5.0). Two milliliters of a filter-sterilized 1% ammonium ferric citrate solution were added to 100 ml medium before pouring the plates. The plates were incubated for 5 days at 30°C and colonies showing beta-glucosidase activity were identified by a brown surrounding halo. The absence of extracellular protease production was determined by replica plating yeast or bacterial colonies onto YPD plates containing 2% casein or onto MRS plates containing 2% casein, respectively. The plates were incubated for 5 days d at 25°C. A clear zone around the colony identified protease activity. Lipase activity was evaluated by replica plating yeast colonies onto agar plates containing 5% peptone, 0.5% glucose, 0.1% NaNO_2_, 0.1% KH_2_PO_4_, 0.1% MgSO_4_, 2% Tween 80, and 0.01% rhodamine B. Lypolitic activity was determined by the presence of a darker halo surrounding the colony which was detectable after 10 days at 25°C. Biogenic amine formation was determined using a modified method of Nikolaou et al. (2006). Yeast and bacteria strains were inoculated on YPD or MRS agar plates, respectively, supplemented with bromocresol purple 0.006 and 1% (w/v) of the amino acids histidine, tyrosine, phenylalanine, tryptophan, lysine, leucine, and arginine. The plates were incubated at 25°C for 3–4 days and growth was examined daily. At the beginning, a yellow halo was observed around the colonies because of glucose fermentation, followed by pH reduction, while in the case of amino acid decarboxylation a purple halo appeared.

### LAB and yeast molecular identification

Total genomic DNA from the yeast strains was prepared according to the method used by Querol et al. (1992) and diluted to 50 ng/μl. ITS1-5,8S-ITS2 region was amplified with primers ITS1 (5′-TCC GTA GGT GAA CCT GCG G-3′) and ITS4 (5′-TCC TCC GCT TAT TGA TAT GC-3′) (White et al., 1990). The polymerase chain reaction (PCR) conditions were as described by Esteve-Zarzoso et al. (1999) with the following modifications: initial denaturation at 94°C for 5 min, followed by 40 cycles consisting of 30 s at 94°C, 30 s at 52°C and 1 min at 72°C, followed by a final extension at 72°C for 10 min and subsequently cooled to 8°C. The amplified DNA products were visualized by agarose gel electrophoresis. DNA from pelleted bacterial cells, grown for 3–5 days at 25°C under vacuum in liquid culture media, was extracted as described by Wilson (2001). The nearly full-length 16S rRNA gene was amplified for all isolates by using the Universal 16S forward primer (5′-GGAGAGTTAGATCTTGGCTCAG-3′), and Universal 16S reverse primer (5′-AGAAAG GAGGTGATCCAGCC-3′). A reaction mixture (50 μl) containing 1 μl (50 ng/μl) genomic DNA, 10× PCR buffer (Euroclone), 2 mM MgCl_2_, 200 μM each dATP, dTTP, dCTP and dGTP, primers Universal 16S forward and reverse, 0.5 μM each, and 1 U DNA polymerase (Euroclone, Italy) was prepared. Genomic DNA was amplified with a 2-min denaturation step at 94°C, followed by 35 cycles of 30 s denaturation at 94°C, 30 s primer annealing at 55°C and 1 min DNA chain extension at 72°C. The PCR was completed by 5 min DNA chain extension at 72°C. DNA sequencing was performed as previously described (Bleve et al., 2011). The obtained sequences, corresponding to a total of 194 yeasts and to 140 bacteria isolates were all identified by a database similarity search in the GENBANK Collection using the BLAST software (http://www.ncbi.nlm.nih.gov/BLAST/).

### HPLC analyses

HPLC analyses of phenolic compounds in the brine were achieved by direct injection of filtered (through a 0.45 μm filter) brine into the chromatographic system. The HPLC apparatus consisted of an Agilent 1100 equipped with a photodiode array detector. The wavelengths used for quantification of phenol compounds were 280, 295, and 320 nm. Separation was achieved according to Li et al. (2008) with some modifications using a Phenomenex Luna 5 μC18 (2) 100 Å column (250 × 4.6 mm), with the temperature of the column set to 30°C. A gradient elution program was utilized with a mobile phase consisting of acetonitrile (solution A) and 1% (v/v) phosphoric acid in water (solution B) as follows: isocratic elution, 5% B, 0–30 min; linear gradient from 5 B to 15% B, 30–50 min; linear gradient from 5 B to 50% B, 50–55 min; linear gradient from 50 B to 100% B, 55–65 min; post time, 10 min before the next injection. The flow rate during the mobile phase was 1.0 ml/min, and the injection volume was 20 μl. All phenol compounds were quantified using calibration curves of authentic phenolic standards. Quantification of sugars, organic acids and alcohols in the brine was achieved by directly injecting the brine (filtered as stated above) into the chromatographic system (Agilent 1100) equipped with a RID-10A refractive index detector (for sugar and alcohol analysis) and with a photodiode array detector set to 210 nm (for the analysis of organic acids). Sugars, organic acids, and alcohols were simultaneously analyzed, according to De Benedictis et al. (2011), on an Aminex HPX-87H column (300 × 7.8 mm) (Bio-Rad) and kept at 55°C. The analytical conditions used were as follows: flow 0.3 ml/min, eluent 0.045 N H_2_SO_4_ with 6% acetonitrile (v/v).

### Volatile compound extraction from olive fruit and brines

Volatile compounds of fermentations were identified by Solid-Phase Micro-Extraction technique in Head Space followed by Gas Chromatography/Mass Spectrometry (HS-SPMEGC/MS) (Pawliszyn, 1997). Analysis of samples was carried out by homogenizing 5 g of stoned drupes and transferring into 20-ml vials covered with a polytetrafluoroethylene (PTFE)/silicone rubber septum containing a micro stirring bar. 8 μL of 4-methyl-2-pentanol in methanol (final concentration 50 μg/ml) were used as an internal standard. The 65 μm DVB/CAR/PDMS coated fiber (Supelco, Spain) was used according to Malheiro et al. (2011). The vials were heated to a controlled temperature (40 ± 0.5°C) in order to reach equilibrium. On the basis of preliminary tests, 30 min exposure time proved suitable for fiber saturation and for reproducibility of the extraction procedure. Before the first daily analysis, the fiber was conditioned in the injector for 10 min at 250°C to remove any volatile contaminants. Fibers were desorbed in a split/splitless injector at 250°C for 5 min. All samples were analyzed in triplicate. For the analysis of volatile compounds in olive brines, the fiber used was a CAR/PDMS-75 μm (needle length 1 cm, needle size 24 ga) (Supelco, Spain). The headspace SPME sampling conditions used were as follows: 10 ml of brine and 0.5 g NaCl were transferred into a 20 ml glass vial and spiked with 20 μL of a 4-methyl-2-pentanol (final concentration 50 μg/ml). The vial was tightly capped with a PTFE-faced silicone septum. The sample was equilibrated for 15 min at 35°C, and then the fiber was exposed to the headspace for 30 min under the same conditions. Desorption of volatiles took place in the injector of the GC/MS for 5 min. Before the first daily analysis, the fiber was conditioned in the injector for 10 min at 250°C to remove any volatile contaminants. All samples were analyzed in triplicate.

### GC/MS analysis

HS-SPME analyses were performed using an AGILENT 6890N gas chromatograph coupled to an AGILENT 5973 mass spectrometer (Agilent, USA). Helium was used as a carrier gas at a constant flow rate of 1 ml/min. The injection port was equipped with an SPME liner (0.75 × 6.35 × 78.5 mm). Separation of compounds was performed on a DB-WAX column (60 m, 0.25 mm i.d., 0.25 mm film thickness, Agilent). The injections were performed in splitless mode. Oven temperature was maintained at 40°C for 5 min, programmed at 3°C/min to 150°C for 20 min. The mass spectrometer was operated in electron impact mode with the electron energy set to 70 eV and a scan range of 30-350 m/z. The temperature of MS source and quadrupole were set to 230 and 150°C. Analyses were performed in full-scan mode. Compounds were identified by comparing the retention times of the chromatographic peaks with those of authentic standards analyzed under the same conditions and by comparison of the retention indices (as Kovats indices) with literature data. MS fragmentation patterns were compared with those of pure compounds, and a mass spectrum database search was performed using the National Institute of Standards and Technology (NIST) MS 98 spectral database. Semi-quantitative determination was carried out by the internal standard method (IS, 4-methyl-2-pentanol). The volatile compounds were quantified by comparison of peak areas to those of internal standards.

### Statistical analysis

Chemical data were subjected to One-Way factor analysis of variance (ANOVA). Significant differences were separated using the Duncan test. The level of significance was set at *P* < 0.05. The comparison of volatile classes of compounds during fermentation was achieved by principal component analysis (PCA). All statistical analyses were carried out using the STATISTICA 7.0 software (StatSoft software package, Tulsa, OK, USA).

## Results

### Population dynamics of the microbiota associated to olives fermentations

**Figure 1:**
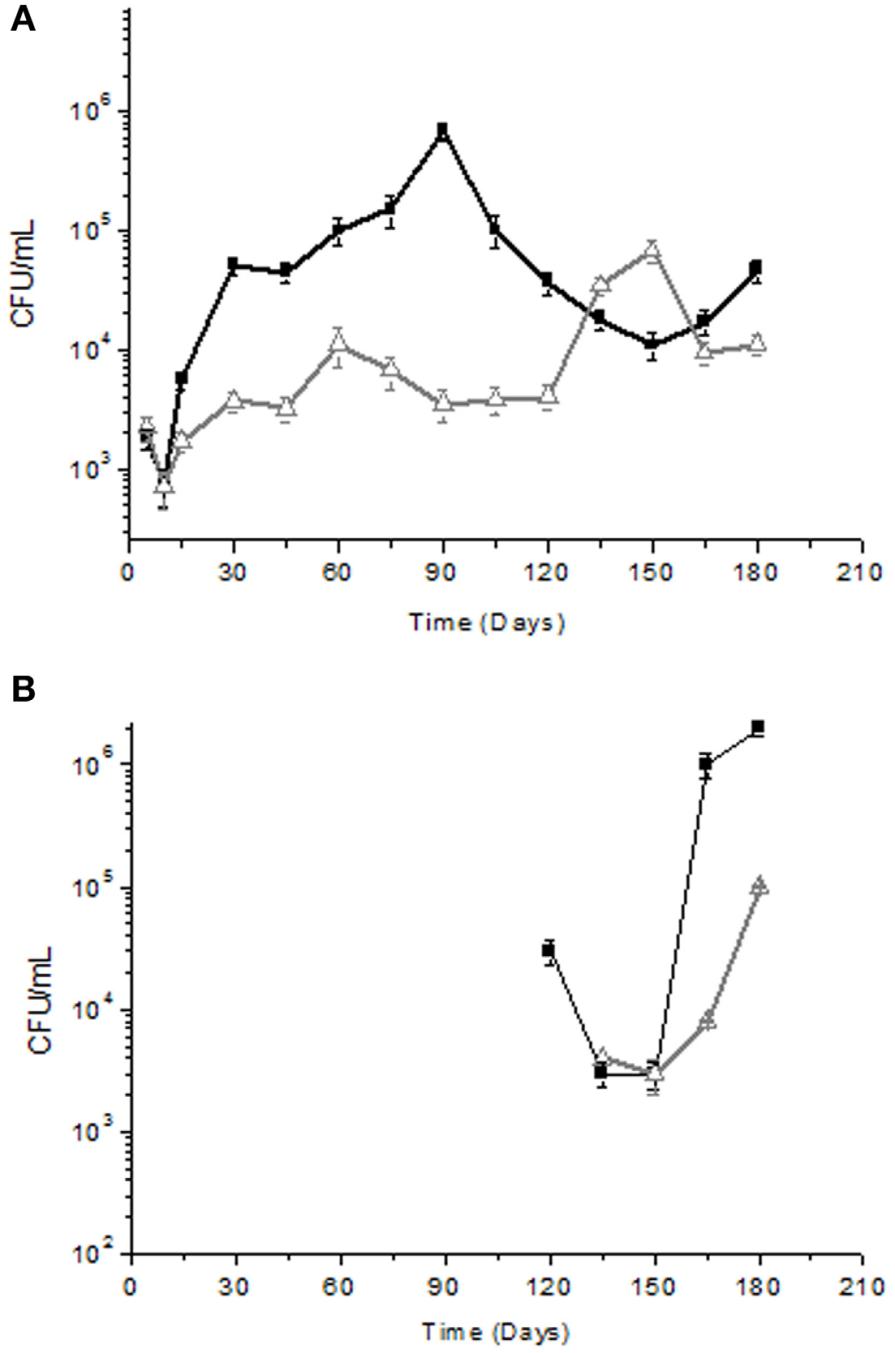
**Yeast (A) and LAB (B) total counts (Log CFU/ml) of Cellina di Nardò** 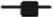 **and Leccino** 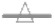 **naturally fermented table olives**. Detection limit ± 10 cfu/ml. LAB were not detected during the period of time between 15 and 120 days of fermentation.

The microbial count values obtained by analyzing the yeast associated with the fresh fruit surface of the two cultivars (Cellina di Nardò and Leccino) was similar, i.e., 6.4 × 10^4^ CFU/g. At the same sampling time, LAB and *Enterobacteriaceae* in Cellina di Nardò were 5.4 × 10^2^ CFU/g and 6.4 × 10^3^ CFU/g (data not shown), respectively. In Leccino table olives, LAB were present at a concentration of 3.3 × 10^2^ CFU/g, while *Enterobacteriaceae* were not detectable. Olives were then subjected to fermentation and brine samples were collected at the specific time intervals. *Enterobacteriaceae* were detected only in olives and brines derived from the Cellina di Nardò cultivar until the 5th day of fermentation and then they resulted undetectable (data not shown). Yeast population showed a decrease during the first 15 days of fermentation and then increased from 10^3^ CFU/ml to 10^4^–10^5^ CFU/ml in both Cellina di Nardò and Leccino. However, yeasts in Cellina di Nardò brines showed a continuous increase in total count until the 90th day of fermentation and then a slow decrease until the 150th day and a new limited increase at the 180th day. By contrast, in Leccino brines, yeast populations showed a rapid increase at the 150th day (6.7 × 10^4^ CFU/ml). The number of viable cells dropped rapidly at the 180th day (9.3 × 10^3^ CFU/ml) of fermentation (Figure 1A).

The dominant species during fermentation time were *Debaryomyces hansenii, Wickerhamomyces anomalus* and *Pichia membranifaciens* for Cellina di Nardò, whereas *Saccharomyces cerevisiae* and *P. membranifaciens* for Leccino olives (Table 1). In particular, the following yeast species were identified in Cellina di Nardò olives: *D. hansenii* (18%), *W. anomalus* (40%), *P. membranifaciens* (28%), *Debaryomyces carsonii* (5%), and *Candida tartarivorans* (4%). From Leccino fermentations, yeast isolates identified belonged to *S. cerevisiae* (51%), *P. membranifaciens* (20%), *Debaryomyces etchellsii* (3%)*, Candida boidinii* (3%), and *Zygosaccharomyces mrakii* (11%). When the identity percentage of the obtained sequences resulted <99%, the isolates were identified only at genus level (*Debaryomyces* sp. and *Pichia* sp. obtained from Cellina di Nardò and of *Candida* sp., *Debaryomyces* sp., *Pichia* sp. and *Zygosaccharomyces* sp. from Leccino). Additional molecular tests (i.e., by RFLP, 5.8 ITS or sequencing D1–D2 domains 26S) will be further performed for their unequivocal identification at species level.

**Table 1:** **Yeast isolates identified during spontaneous fermentation of Cellina di Nardò and Leccino olives**.

	**Time (day)**	**Species**	**Number of isolates**	**Percentage (%)**	**Sum of isolates**		**Time (day)**	**Species**	**Number of isolates**	**Percentage (%)**	**Sum of isolates**
Cellina di Nardò	0	*Debaryomyces carsonii*	2	10	20	Leccino	0	*Candida boidinii*	4	20	20
		*Debaryomyces hansenii*	14	70				*Candida* sp.	4	20	
		*Debaryomyces* sp.	2	10				*Saccharomyces cerevisiae*	12	60	
		*Wickerhamomyces anomalus*	2	10			15	*Candida boidinii*	5	13	40
	15	*Debaryomyces carsonii*	4	10	40			*Saccharomyces cerevisiae*	26	65	
		*Debaryomyces hansenii*	27	68				*Zygosaccharomyces mrakii*	6	15	
		*Debaryomyces* sp.	9	22				*Zygosaccharomyces* sp.	3	7	
	30	*Debaryomyces hansenii*	21	53	40		30	*Candida* sp.	2	5	40
		*Debaryomyces* sp.	3	7				*Saccharomyces cerevisiae*	29	73	
		*Pichia* sp.	1	2				*Zygosaccharomyces mrakii*	7	17	
		*Wickerhamomyces anomalus*	15	38				*Zygosaccharomyces* sp.	2	5	
	45	*Debaryomyces carsonii*	3	7	40		45	*Candida boidinii*	2	5	40
		*Debaryomyces hansenii*	19	48				*Saccharomyces cerevisiae*	28	70	
		*Pichia* sp.	2	5				*Zygosaccharomyces mrakii*	6	15	
		*Wickerhamomyces anomalus*	16	40				*Zygosaccharomyces* sp.	4	10	
	60	*Debaryomyces hansenii*	11	28	40		60	*Pichia membranifaciens*	3	7	40
		*Debaryomyces* sp.	4	10				*Saccharomyces cerevisiae*	26	65	
		*Pichia membranifaciens*	5	12				*Zygosaccharomyces mrakii*	11	28	
		*Wickerhamomyces anomalus*	20	50			75	*Pichia membranifaciens*	2	5	40
	75	*Debaryomyces carsonii*	8	20	40			*Saccharomyces cerevisiae*	27	67	
		*Pichia membranifaciens*	4	10				*Zygosaccharomyces mrakii*	5	13	
		*Wickerhamomyces anomalus*	28	70				*Zygosaccharomyces* sp.	6	15	
	90	*Debaryomyces carsonii*	4	10	40		90	*Pichia membranifaciens*	3	7	40
		*Pichia membranifaciens*	11	27				*Saccharomyces cerevisiae*	28	70	
		*Wickerhamomyces anomalus*	25	63				*Zygosaccharomyces mrakii*	8	20	
	105	*Pichia membranifaciens*	10	25	40			*Zygosaccharomyces* sp.	1	3	
		*Pichia* sp.	3	8			105	*Pichia membranifaciens*	5	13	40
		*Wickerhamomyces anomalus*	27	67				*Saccharomyces cerevisiae*	24	60	
	120	*Pichia membranifaciens*	11	27	40			*Zygosaccharomyces mrakii*	11	27	
		*Wickerhamomyces anomalus*	29	73			120	*Pichia membranifaciens*	25	63	40
	135	*Pichia membranifaciens*	16	40	40			*Saccharomyces cerevisiae*	15	37	
		*Wickerhamomyces anomalus*	24	60			135	*Pichia membranifaciens*	18	45	40
	150	*Candida tartarivorans*	8	20	40			*Pichia* sp.	6	15	
		*Debaryomyces carsonii*	4	10				*Saccharomyces cerevisiae*	16	40	
		*Pichia membranifaciens*	22	55			150	*Candida* sp.	7	17,5	40
		*Wickerhamomyces anomalus*	6	15				*Debaryomyces ethcellsii*	2	5	
	175	*Candida tartarivorans*	6	15	40			*Debaryomyces* sp.	5	12,5	
		*Pichia membranifaciens*	31	77				*Pichia membranifaciens*	16	40	
		*Wickerhamomyces anomalus*	3	8				*Saccharomyces cerevisiae*	10	25	
	180	*Candida tartarivorans*	6	15	40		175	*Candida boidinii*	3	8	40
		*Pichia membranifaciens*	32	80				*Candida* sp.	7	17,5	
		*Wickerhamomyces anomalus*	2	5				*Debaryomyces ethcellsii*	7	17,5	
								*Debaryomyces* sp.	8	20	
								*Pichia membranifaciens*	9	22	
								*Saccharomyces cerevisiae*	6	15	
							180	*Candida boidinii*	1	2,5	40
								*Debaryomyces ethcellsii*	5	12,5	
								*Debaryomyces* sp.	8	20	
								*Pichia membranifaciens*	18	45	
								*Saccharomyces cerevisiae*	8	20	

Concerning LAB detection, the results obtained are reported in Figure 1B. In Cellina di Nardò after the starting time, LAB were no more detectable until the 120th day of fermentation (3.2 × 10^4^ CFU/ml) and then they increased (2 × 10^6^ CFU/ml) until the 180th day of fermentation. In Leccino cultivar, LAB were detectable (3.9 × 10^3^ CFU/ml) at the 135th day of fermentation and then they increased up to 1.4 × 10^5^ CFU/ml at the 180th day of fermentation. Bacterial isolates obtained from Cellina di Nardò fermentation belonged to species *L. plantarum* (68%), *Kocuria* spp. (5%), and *Swaminathania salitolerans* (13%). The bacterial isolates identified at species level (sequence identity percentage > 98%) from Leccino fermentation belonged to the *L. plantarum* (89%). Identification at species level of *Lactobacillus* sp. isolates obtained from Cellina di Nardò and Leccino, is now forthcoming by using multiplex PCR of recA gene (Torriani et al., 2001). Moreover, although LAB were not detectable until the 120th and 135th day of fermentation for Cellina di Nardò and Leccino, respectively, the dominant species was *L. plantarum* for both the table olive cultivars (Table 2).

**Table 2:** **Bacteria isolates identified during spontaneous fermentation of Cellina di Nardò and Leccino olives**.

	**Time**	**Species**	**Number of isolates**	**Percentage (%)**	**Sum of isolates**
**Cellina di Nardò**	0	*Kocuria*	12	100	12
	120	*Swaminathania salitolerans*	26	100	26
	135	*Lactobacillus plantarum*	35	77	52
		*Lactobacillus* sp.	12	13	
		*Swaminathania salitolerans*	5	10	
	150	*Lactobacillus plantarum*	40	88	50
		*Lactobacillus* sp.	10	12	
	175	*Lactobacillus plantarum*	50	100	50
	180	*Lactobacillus plantarum*	38	94	50
		*Lactobacillus* sp.	12	6	
**Leccino**	0	*Lactobacillus plantarum*	12	100	12
	135	*Lactobacillus plantarum*	40	77	52
		*Lactobacillus* sp.	12	23	
	150	*Lactobacillus plantarum*	39	75	52
		*Lactobacillus* sp.	13	25	
	175	*Lactobacillus plantarum*	52	100	52
	180	*Lactobacillus plantarum*	52	100	52

### Yeast strains technological characterization

A dedicated procedure was assessed for the isolation and technological characterization of yeasts and bacteria from fermented table olives. As a first step we set up the formulation of two media, for the evaluation of yeasts and LAB technological properties. Both media reproduced the severe physico-chemical constraints usually found into the fermenting brines. Isolated microorganisms were assayed for their ability to grow in a synthetic model brine (MB), that varied in their composition throughout the fermentations, to be used as a reliable and reproducible substrate for the *in vitro* technological assays. To achieve the formulation of the model brines, brine samples deriving from several fermentations of different black olive cultivars were analyzed by reversed-phase HPLC-DAD to determine their chemical profile, in particular, that corresponding to the phenolic compounds (Bleve et al., 2015). Five hundred yeast isolates for each of the two olive cultivars were applied on MB and incubated for 15 days at 15°C. This temperature represented the average value observed during late autumn and winter period when Leccino and Cellina di Nardò olives were generally fermented. Out of 500 initial yeast isolates, 278 isolates from Cellina di Nardò and 101 isolates from Leccino fermentations were able to survive the above constraints. In the second step of the proposed procedure, the microbial strains were tested by plate assays in order to evaluate (i) the presence of beta-glucosidase activity, required to degrade oleuropein, and (ii) the inability to produce biogenic amines. Afterward, 77 yeast isolates from Cellina di Nardò and 53 from Leccino demonstrated that they were able to satisfy the above parameters. These isolates were further evaluated for the presence of protease and lipase activities (not shown). For beta-glucosidase activity it was assigned a score 3 (intense brown), 2 (light brown), 1 (yellow-milky), 0 (white), whereas, for amino acids decarboxylation activities, the value 3 was considered for isolates surrounded by an intense purple halo, 2 for isolates which produced intense blue halo, 1 for isolates producing slight blue halo and the value 0 was assigned for isolates that remained white. The yeast population characterized by intense beta-glucosidase activity (score 3) and absence of amino acids decarboxylation activities (score 0) consisted of 77 Cellina di Nardò isolates and 53 Leccino isolates. These isolates were then evaluated for the presence of protease and lipase activities: 17 isolates from Cellina di Nardò and 11 isolates from Leccino showed protease activity on plate against casein, whereas none of the isolates deriving from the second selection step demonstrated lipase activity. The third step in the proposed protocol consisted of the identification at species level of the yeast isolates that did not showed protease activity by PCR analysis of their rRNA genes.

At the end of this procedure, from Cellina di Nardò one yeast isolate was selected for the species *P. anomala*, identified by the Accession number LK392318, one isolate of *D. hansenii* (Accession number LK322319), one for the species *P. membranifaciens* (Accession number LK322320), whereas, it was selected from Leccino one isolate of *S. cerevisiae* (Accession number LK392321) and one of *P. membranifaciens* (Accession number LK392322).

### LAB technological characterization

As decribed by Bleve et al. (2015) the selection of bacterial isolates was carried out on the MB *ad hoc* formulated. After this step, the bacterial population resistant to the above constraint was isolated, consisting of 110 (out of 240) isolates from Cellina di Nardò and 90 (out of 220) isolates from Leccino. The presence of beta-glucosidase and amino acids decarboxylation activities were tested as described above. After this second selection step, the LAB population which satisfied the above parameters (intense beta-glucosidase activity, score 3, and absence of amino acids decarboxylation activities, score 0) consisted of 18 Cellina di Nardò isolates and 14 Leccino isolates. These isolates were then evaluated for the presence of protease and lipase activities. None of the isolates from Cellina di Nardò isolates and Leccino showed protease and lipase activities.

At the end of this procedure, two LAB isolates both belonging the species *L. plantarum* were selected from Cellina di Nardò and Leccino and identified by the Accession number LK392323 and LK392324, respectively.

### Chemical dynamics of brines

**Figure 2:**
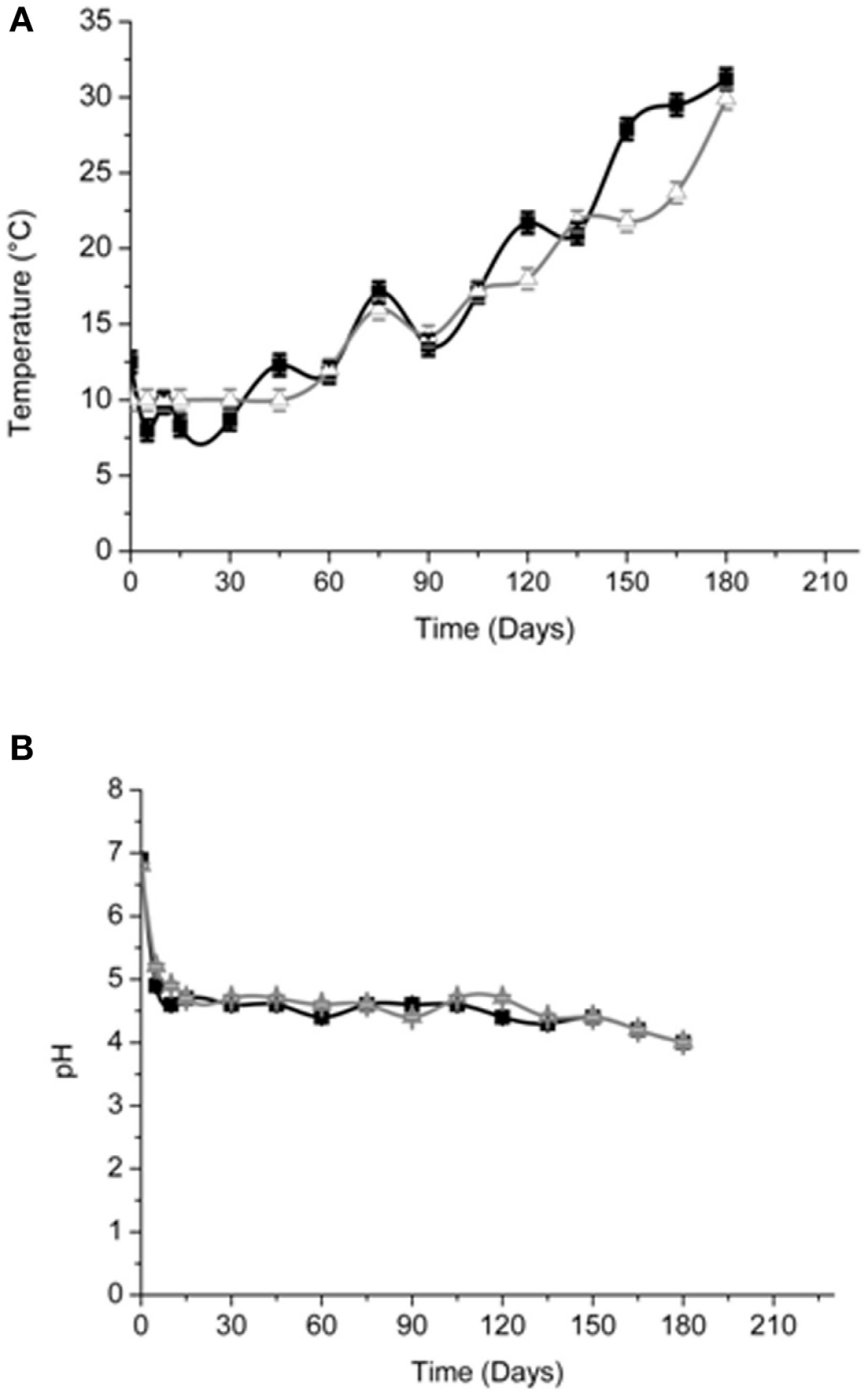
**Temperature (A) and pH (B) of Cellina di Nardò** 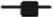 **and Leccino** 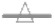 **naturally fermented table olives**.

**Figure 3:**
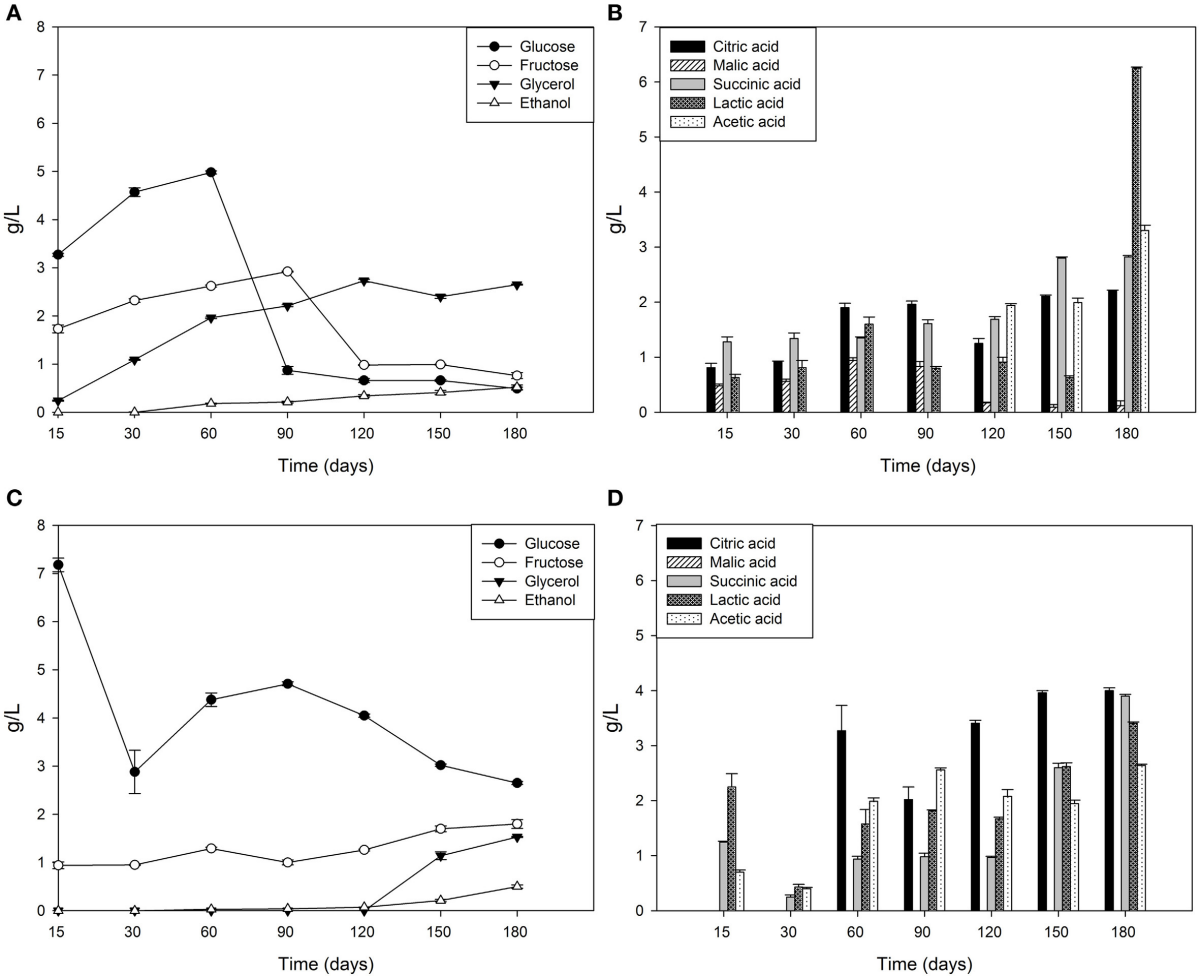
**Sugar, organic acid and alcohol evolution in Cellina di Nardò (A,B) and Leccino (C,D) brines during natural fermentation process**.

**Figure 4:**
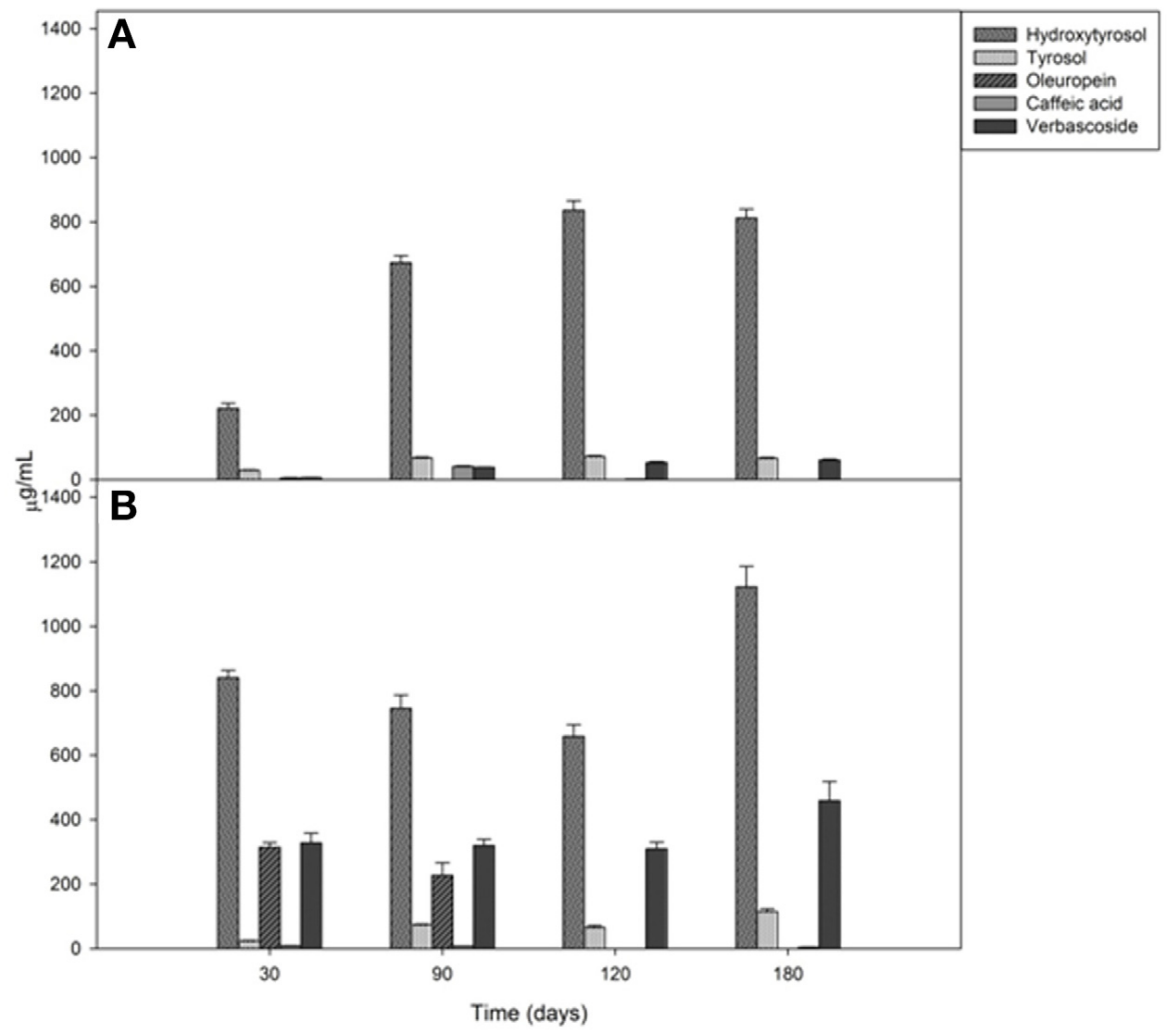
**Mono and polyphenol compound concentrations in Cellina di Nardò (A) and Leccino (B) brines during natural fermentation process**.

Brine temperatures increased from 8 to 17°C during the first 3 months of fermentation and then it reached 30°C at the 180th day (Figure 2A). The pH values quickly decreased (4.4–4.7) within the first 5–10 days of fermentation and, after the 120th, they declined at its minimum values (Figure 2B). Salinity values were checked throughout the fermentation to maintain it almost stable about a value of 10% (w/v) (data not shown). Glucose concentrations increased until the 30th and the 60th of fermentation respectively, in Cellina di Nardò and Leccino samples and then they decreased until the end of the process (Figure 3A). The fructose content should apparently be a constant increase during the fermentation of Leccino table olives, whereas in Cellina di Nardò brines, after a substantial increase during the first 90 days, fructose levels decreased to a final concentration of about 0.7 g/L (Figures 3A,C). The level of total organic acids in Leccino brines was found to be higher than in Cellina di Nardò and these compounds increased during fermentation (Figures 3B,D). Ethanol concentration increased gradually with time and reached a final concentration of about 0.5 g/L (Figures 3A,C). Figure 4 shows the concentration dynamics of the following simple phenolic compounds in the brine: hydroxytyrosol, oleuropein, and tyrosol. In general, Leccino brine samples showed a greater amount of phenolic compounds than Cellina di Nardò brines. In all samples, the main phenolic compound detected during fermentation was hydroxytyrosol, it being about 200 and 800 μg/ml, respectively in Cellina di Nardò and Leccino brines. Oleuropein was undetectable in Cellina di Nardò brines soon after the 30th day of fermentation (Figure 4A) whereas, in Leccino brines, it was observed until 90 days of fermentation (Figure 4B). Tyrosol concentration respectively, increased in the first 60 and 90 days of fermentation in Cellina di Nardò and Leccino brines and then it remained constant until the end of process (Figures 4A,B).

### Analysis of volatile compounds in olives and brines

The analytical SPME/GC–MS method used in this work allowed the correct identification and quantification of 54 and 44 volatile compounds in Cellina di Nardò and Leccino olives and brines, respectively. All the volatile compounds identified were grouped into aldehydes/ketones, esters, alcohols, hydrocarbons, volatile phenols, fatty acids, monoterpenes, and lactones classes. Aldehydes and ketones content was significantly different in the two olive cultivars, ranging from 88.61 ± 7.25 μg/kg in Cellina di Nardò olives and 104.25 ± 6.01 μg/kg in Leccino olives at 30 days. After 180 days of fermentation they resulted 30.26 ± 4.21 and 61.93 ± 4.09 μg/kg for Cellina di Nardò and Leccino at, respectively (Table 3). With the exception of benzaldehyde, all identified aldehydes showed a decrease in concentration during fermentation. Alcohol and ester contents increased during fermentation, with higher concentrations in Cellina di Nardò than in Leccino (Table 3). In fact, the levels of ethyl acetate, isoamyl acetate, ethyl hexanoate (green apple aroma), and ethyl octanoate (sweet aroma) increased during fermentation in both table olive cultivars (Table 3). Short chain fatty acids (acetic, propanoic, 2-methylpropanoic) were detected in both fermentations, with acetic acid being the major representative of this group. Acetic acid was present throughout the fermentation period and in both cultivars and is representative of yeast and LAB metabolism following oxidation of ethanol (Table 3). Levels of acetic acid increased progressively from 7.73 ± 0.04 to 59.32 ± 1.30 μg/kg in Cellina di Nardò olives, and from 5.22 ± 0.30 to 25.06 ± 0.93 μg/kg in Leccino olives. In both fermentations, total amounts of hydrocarbons (octane, toluene, styrene, trimethyl benzene, minor volatile compounds) increased from the initial to the middle stages of fermentation and then a slight decrease during the final period (Table 3). Terpene concentrations also increased during fermentation in both table olive cultivars. There were five different terpenes, of which 3 were monoterpenes (linalol, linalolox, 3,7 dimethyl- 2,6 octadienal) and two sesquiterpenes (cymene and α-pinene). The highest amounts of all these terpenes were detected in Cellina di Nardò olives (Table 3). The principal free volatile classes showed different distributions in olives from brines (Table 4). In fact, in Cellina di Nardò brines collected after 90 and 180 days of fermentation, a greater concentration of esters, alcohols and short chain fatty acids was observed than in the corresponding drupes. The most representative ester species was ethyl acetate, followed by methyl acetate. Propionic esters were detected in the last fermentation stage only in Cellina di Nardò brine. Alcohols detected in higher concentrations in Cellina di Nardò brines were 2+3-methyl-1-butanol, followed by 1-propanol and cis 3 hexen-ol (Z), whereas in Leccino brines there was a higher concentration of hexan-1-ol, followed by 2+3-methyl-1-butanol and heptanol. Analogously to what observed in drupes, in Cellina di Nardò brines there was a higher amount of fatty acids than in Leccino brines.

**Table 3:** **SPME/GC–MS quantitative data, including concentration (μg/kg) with standard deviation (SD) of all the volatile compounds identified in Cellina di Nardò and Leccino table olives**.

**Compounds**	**Cellina di Nardò table olives**			**Leccino table olives**			**Odor descriptors**
	**30 days**	**90 days**	**180 days**	**30 days**	**90 days**	**180 days**	
	mean ± SD μg/kg^*^	mean ± SD μg/kg^*^	mean ± SD μg/kg^*^	mean ± SD μg/kg^*^	mean ± SD μg/kg^*^	mean ± SD μg/kg^*^	
**ALDEHYDES-KETONS**							
2-methylpropanal	13.16 a ± 0.90	ND	ND	16.54 b ± 0.80	ND	ND	cooked-caramel
2-methylbutanal	17.19 c ± 0.20	9.37 b ± 0.60	7.12 a ± 0.14	23.66 d ± 0.91	14.13 b ± 0.23	13.70 b ± 0.22	malty
3-methylbutanal	14.80 b ± 0.55	8.53 a ± 1.02	7.51 a ± 0.75	31.50 c ± 2.20	20.41 b ± 2.11	17.65 b ± 0.45	malty
Hexanal	10.51 b ± 2.02	5.70a ± 1.40	ND	ND	ND	ND	green-sweet-green apple
2,6-dimethyl-4-heptanon	14.40 b ± 2.20	7.33 a ± 0.77	ND	10.30ab ± 0.05	12.51b ± 1.23	8.05a ± 1.80	oily-fatty-woody
Heptanal	4.50 b ± 0.05	3.15 a ± 0.21	ND	4.49b ± 0.20	ND	ND	tallowy-pungent
Nonanal	11.90 b ± 1.30	16.92 b ± 0.85	9.1 a ± 3.12	6.64 a ± 0.85	9.77 a ± 1.22	10.43 a ± 0.20	fatty-waxy-pungent
Benzaldehyde	2.20 ab ± 0.03	2.90 bc ± 0.40	3.43 c ± 0.20	2.32 ab ± 0.20	1.82 a ± 0.04	3.35 c ± 0.60	almond
trans 2-decenal	ND	ND	3.12a ± 0.1	8.80b ± 0.80	ND	8.55b ± 0.82	painty-fishy-fatty
Total amounts	88.61 ± 7.25	53.87 ± 5.25	30.26 ± 4.21	104.25 ± 6.01	58.64 ± 2.83	61.93 ± 4.09	
**Esters**							
Methyl acetate	ND	4.95 a ± 0.61	15.30 b ± 0.60	ND	ND	14.12 b ± 1.52	Ethereal, sweet
Ethyl acetate	9.90 a ± 0.52	31.99 b ± 0.62	102.20 d ± 4.80	ND	13.80 ab ± 3.11	57.54 c ± 2.60	sticky–sweet
Ethyl propanoate	ND	ND	38.41 a ± 0.74	ND	ND	ND	fruity-strong
2 methyl ethyl propanoate	ND	ND	7.71 a ± 0.50	ND	ND	ND	
n-propyl acetate	ND	ND	19.5 a ± 0.43	ND	ND	ND	
Isobutyl acetate	ND	ND	12.80 a ± 0.80	ND	ND	ND	
Ethyl butanoate	ND	ND	5.12 a ± 0.83	ND	ND	5.01 b ± 0.50	sweet-fruity
Propyl propanoate	ND	ND	11.88 a ± 0.61	ND	ND	ND	
Ethyl 2-methyl butanoate	ND	ND	11.50 ± 0.90	3.80 ± 0.04	3.90 ± 0.32	5.22 ± 0.24	fruity
Ethyl 3-methyl butanoate	ND	ND	10.96 c ± 0.93	4.20 a ± 0.40	4.52 a ± 0.04	5.60 b ± 0.31	fruity
Isoamyl acetate	ND	9.40 b ± 0.91	54.20 d ± 2.20	ND	2.36 a ± 0.11	16.72 c ± 5.32	
3 methyl 1 butanol propanoate	ND	ND	9.00 c ± 0.43	ND	4.84 a ± 0.24	5.20 b ± 0.50	
2-methyl 3-butenoic acid ethyl ester	ND	ND	2.80 a ± 0.31	ND	ND	ND	
Ethyl hexanoate	ND	4.86 a ± 0.73	8.76 ab ± 0.70	ND	6.10 b ± 1.11	12.35 c ± 1.09	
Hexyl acetate	ND	1.58 a ± 0.04	7.70 c ± 0.20	ND	ND	3.55 b ± 0.50	sweet-fruity-floral
Ethyl 3-hexenoate	ND	ND	0.74a ± 0.05	ND	4.11b ± 0.43	4.60b ± 0.51	
3-hexenol acetate	ND	ND	15.20 a ± 0.43	ND	ND	ND	
Ethyl heptanoate	ND	1.35 a ± 0.1	1.55 b ± 0.14	ND	ND	ND	
Ethyl octanoate	ND	1.75 a ± 0.24	3.72 b ± 0.50	ND	10.14 c ± 1.22	12.30 d ± 0.02	
Benzyl acetate	ND	ND	2.82 a ± 0.30	ND	ND	2.90 a ± 0.16	
Methyl salicylate	ND	ND	4.12 a ± 0.06	ND	ND	ND	
Ethyl 2-hydroxy-benzoate	ND	ND	2.45 a ± 0.04	ND	ND	2.50 a ± 0.17	
Phenyl ethyl acetate	ND	ND	4.56 b ± 0.12	ND	ND	ND	
Total amounts	9.90 ± 0.52	55.87 ± 3.25	352.97 ± 16.62	7.98 ± 0.44	49.81 ± 6.58	147.61 ± 13.44	
**ALCOHOLS**							
1-propanol	ND	ND	19.95 b ± 0.84	ND	ND	5.27 a ± 0.60	
2 methyl-1-propanol	2.11 a ± 0.80	13.96 c ± 1.10	9.43 b ± 0.80	ND	7.74 b ± 0.54	8.01 b ± 0.33	Penetrating, solvent
2+3-methyl-1-butanol	10.7 a ± 0.20	86.80 c ± 6.02	60.21 b ± 2.70	10.30 a ± 0.41	49.95 b ± 2.11	46.20 b ± 1.24	woody-whiskey-sweet
3 methyl-3-buten-ol	ND	ND	1.60 a ± 0.07	NDa	ND	ND	
Hexanol	13.40 bc ± 1.10	17.50 de ± 1.32	20.20 e ± 1.21	8.76 a ± 0.72	11.26 ab ± 1.03	15.00 cd ± 1.80	fruit-banana
trans 3-hexen-ol (E)	ND	ND	1.21 a ± 0.24	ND	ND	ND	green
cis 3-hexen-ol (Z)	15.43 b ± 0.55	25.26 c ± 0.55	50.95 d ± 0.92	1.74 a ± 0.04	1.81 a ± 0.23	2.44 a ± 0.04	green
Heptanol	ND	5.10 a ± 0.50	6.40 b ± 0.25	ND	8.81cd ± 0.90	12.20 d ± 0.25	earthy-sweefy
Nonanol	ND	ND	6.85 a ± 0.10	ND	ND	ND	fatty
Benzylalcohol	ND	7.80 b ± 0.80	ND	ND	2.31 a ± 0.13	8.30 b ± 0.70	
Total amounts	41.64 ± 2.65	156.35 ± 10.29	179.43 ± 7.13	20.80 ± 1.17	81.92 ± 4.94	97.42 ± 4.96	
**HYDROCARBONS**							
Octane	ND	ND	ND	68.50 a ± 0.93	78.90a ± 3.42	71.30 a ± 4.10	
Toluene	6.81 a ± 1.65	ND	ND	ND	ND	ND	
Styrene	ND	43.30 b ± 5.16	31.90 b ± 1.80	10.70 a ± 1.02	89.80 c ± 3.25	77.21 c ± 2.10	
Total amounts	6.81 ± 1.65	43.27 ± 5.16	31.90 ± 1.80	79.17 ± 1.95	168.70 ± 6.67	148.51 ± 6.20	
**FATTY ACIDS**							
Acetic acid	7.73 a ± 0.04	13.25 a ± 0.22	59.32 c ± 1.30	5.22 a ± 0.30	8.7 a ± 1.1	25.06 b ± 0.93	sour-vinegary
Propanoic acid	ND	ND	10.22 a ± 0.32	ND	ND	ND	pungent-sour
2-methyl propanoic acid	ND	ND	1.2 a ± 0.05	ND	ND	ND	
Total amounts	7.73 ± 0.04	13.25 ± 0.22	70.72 ± 1.67	5.22 ± 0.30	8.67 ± 1.1	25.06 ± 0.93	
**VOLATILES PHENOLS**							
Guaiacol	ND	ND	ND	ND	35.55 a ± 3.42	182.20 b ± 1.80	
**TERPENES**							
3,7 dimethyl 1,3,7 octatriene (cimene)	8.01c ± 0.53	10.94 e ± 0.90	9.50 d ± 0.23	2.95 a ± 0.91	5.65 b ± 0.22	4.70 b ± 0.12	
3,7 dimethyl 2,6 octadienal	ND	ND	ND	ND	ND	0.80 a ± 0.20	
α pinene	ND	ND	ND	ND	2.10 a ± 0.15	3.01 b ± 0.40	
Linalolox	ND	ND	3.63 a ± 0.06	ND	ND	ND	
Linalol	ND	ND	1.40 a ± 0.05	ND	ND	ND	
Total amounts	8.01 ± 0.53	10.94 ± 0.90	14.50 ± 0.34	2.95 ± 0.91	7.75 ± 0.37	8.51 ± 0.72	

* *Results expressed as mean ± SD; ND, not detected*.

*For each compound, according to the result of the One-Way ANOVA test, concentration values that do not share a common superscript are significantly different (P < 0.05)*.

**Table 4:** **SPME/GC–MS quantitative data, including concentration (μg/L) with standard deviation (SD) of all the volatile compounds identified in Cellina di Nardò and Leccino brines**.

**Compounds**	**Cellina di Nardò brines**			**Leccino brines**		
	**30 days**	**90 days**	**180 days**	**30 days**	**90 days**	**180 days**
	mean ± SD μg/L^*^	mean ± SD μg/L^*^	mean ± SD μg/L^*^	**mean ± SD μg/L**	mean ± SD μg/L^*^	mean ± SD μg/L^*^
**ALDEHYDES-KETONS**						
2-methyl propanal	2.50 a ± 0.23	ND	ND	ND	ND	ND
2-methyl butanal	7.93 c ± 0.90	ND	ND	4.02 ab ± 0.32	5.70 bc ± 0.20	3.40 a ± 0.20
3-methyl butanal	18.60 c ± 0.43	ND	ND	5.64 b ± 0.51	2.10 a ± 0.9	2.91 ab ± 0.05
Hexanal	1.70 b ± 0.10	ND	ND	0.60 a ± 0.20	ND	ND
Acetoino	ND	ND	13.02 b ± 0.44	ND	ND	ND
Nonanal	ND	1.02 a ± 0.02	ND	1.53 a ± 0.34	7.70 b ± 0.71	ND
Decanal	ND	ND	ND	ND	1.4 b ± 0.10	ND
Benzaldehyde	5.75 b ± 1.05	3.45 b ± 0.10	ND	3.50 b ± 0.25	3.30 a ± 0.40	8.76 c ± 0.84
Total amounts	36.48 ± 2.71	4.47 ± 0.12	13.02 ± 0.44	15.29 ± 1.62	20.20 ± 1.50	15.07 ± 1.09
**ESTERS**						
Methyl acetate	3.76 a ± 0.76	7.82 a ± 1.90	32.71 c ± 1.13	3.50 a ± 0.11	3.24a ± 0.05	16.80 b ± 0.96
Ethyl acetate	33.71 a ± 2.40	174.11 c ± 4.93	226.97 d ± 5.80	21.26 a ± 1.60	25.63 a ± 1.54	114.21 b ± 9.43
Ethyl propanoate	ND	ND	96.97 a ± 2.05	ND	ND	ND
n-propyl acetate	ND	ND	91.70 a ± 5.52	ND	ND	ND
Acetic acid 2 methyl propyl ester	ND	2.78 a ± 0.20	13.45 b ± 0.51	ND	ND	1.64 a ± 0.07
Ethyl butanoate	ND	ND	2.20 a ± 0.10	ND	ND	ND
2 methyl ethyl butanoate	ND	ND	6.96 a ± 0.74	ND	ND	ND
3 methyl ethyl butanoate	ND	ND	1.14 a ± 0.10	ND	ND	ND
Isoamyl acetate	0.92 a ± 0.41	5.04 b ± 0.66	27.22 c ± 1.10	ND	ND	4.55 b ± 1.03
Ethyl hexanoate	ND	1.00 a ± 0.05	0.94 a ± 0.10	ND	ND	ND
Ethyl octanoate	ND	ND	ND	ND	ND	0.90 a ± 0.20
Methyl salycilate	ND	ND	ND	ND	ND	10.92a ± 0.90
3 hexen-1-ol acetate	ND	ND	2.06 b ± 0.95	ND	ND	ND
Ethyl lactate	ND	ND	1.64 c ± 0.72	ND	ND	0.80 b ± 0.10
Total amounts	38.40 ± 3.57	190.75 ± 7.54	503.96 ± 18.82	24.76 ± 1.71	28.87 ± 1.59	149.82 ± 12.60
**ALCOHOLS**						
2 butanol	ND	ND	3.34 a ± 0.10	ND	ND	ND
1 propanol	1.60 a ± 0.11	4.05 a ± 0.21	36.50 b ± 2.40	ND	1.10 a ± 0.10	3.45 a ± 1.01
2 methyl-1-propanol	6.26 a ± 0.70	15.61 b ± 0.71	15.82 b ± 1.65	0.89 a ± 0.12	1.63 a ± 0.10	3.50 a ± 0.44
1 butanol	1.42 ± 0.06	1.32 ± 0.10	1.02 ± 0.03	1.05 ± 0.03	1.27 ± 0.12	4.12 ± 0.60
2+3 methyl-1-butanol	33.60 b ± 0.81	204.33 d ± 3.50	109.66 c ± 5.32	ND	18.25 ab ± 3.20	15.17 ab ± 1.51
Pentan-1-ol	ND	ND	7.07 a ± 1.60	ND	ND	ND
2 penten-ol	ND	0.34a ± 0.04	3.70 b ± 0.60	ND	ND	ND
3 methyl-2-buten-ol	ND	1.06 a ± 0.30	ND	ND	ND	ND
Hexan-1-ol	14.80 ab ± 1.54	31.60 c ± 2.11	19.62 ac ± 2.85	5.80 a ± 0.70	10.14 ab ± 0.10	21.98 ac ± 0.90
trans 3 hexen-ol (E)	ND	0.93 a ± 0.50	1.50 b ± 0.50	ND	ND	ND
cis 3 hexen-ol (Z)	18.66 ab ± 2.96	39.46 bc ± 2.60	65.80 c ± 2.32	11.60 ab ± 0.04	1.48 a ± 0.42	5.42 a ± 1.05
Heptanol	ND	ND	ND	2.80 a ± 0.20	2.41 a ± 0.20	9.53 b ± 0.60
Benzylalcohol	ND	1.75 a ± 0.90	5.65 b ± 0.63	0.68 a ± 0.14	0.5 a ± 0.04	1.06 a ± 0.25
Total amounts	76.34 ± 6.18	300.40 ± 10.96	428.42 ± 18.00	163.03 ± 1.23	145.71 ± 4.38	241.94 ± 6.36
**HYDROCARBONS**						
Trimethyl benzen	1.30 ab ± 0.20	0.64 a ± 0.13	1.20 ab ± 0.32	14.98 c ± 0.22	2.10 ab ± 0.34	2.35 ab ± 0.60
Styrene	ND	1.60 a ± 0.30	0.50 a ± 0.03	2.20 a ± 1.01	24.53 b ± 3.80	ND
Total amounts	1.30 ± 0.20	2.22 ± 0.33	1.70 ± 0.35	17.18 ± 1.23	26.63 ± 4.14	2.35 ± 0.60
**FATTY ACIDS**						
Acetic acid	2.72 a ± 0.10	11.50b ± 2.10	164.30d ± 8.64	1.30a ± 0.24	ND	36.31c ± 3.60
Propanoic acid	ND	ND	35.01 b ± 2.12	ND	ND	ND
2 methyl propanoic acid	ND	0.44a ± 0.10	1.66 b ± 0.05	ND	ND	ND
2 methyl butanoic acid	ND	ND	2.80 c ± 0.13	ND	ND	1.30 b ± 0.16
Total amounts	2.72 ± 0.10	11.93 ± 2.20	203.74 ± 10.94	1.30 ± 0.24		36.31 ± 3.76
**VOLATILES PHENOLS**						
Guaiacol	2.60 a ± 0.41	0.93a ± 0.23	22.91 b ± 0.53	ND	ND	81.51 c ± 3.22
**Terpenes**				ND	ND	
Terpineol	ND	ND	ND	ND	ND	1.10 b ± 0.14
**Lactones**						
Butyrolacton	ND	0.92 c ± 0.15	0.55 b ± 0.14	ND	ND	ND

* *Results expressed as means ± SD; ND, not detected*.

*For each compound, according to the result of the One-Way ANOVA test, concentration values that do not share a common superscript are significantly different (P < 0.05)*.

### Principal component analysis

**Figure 5:**
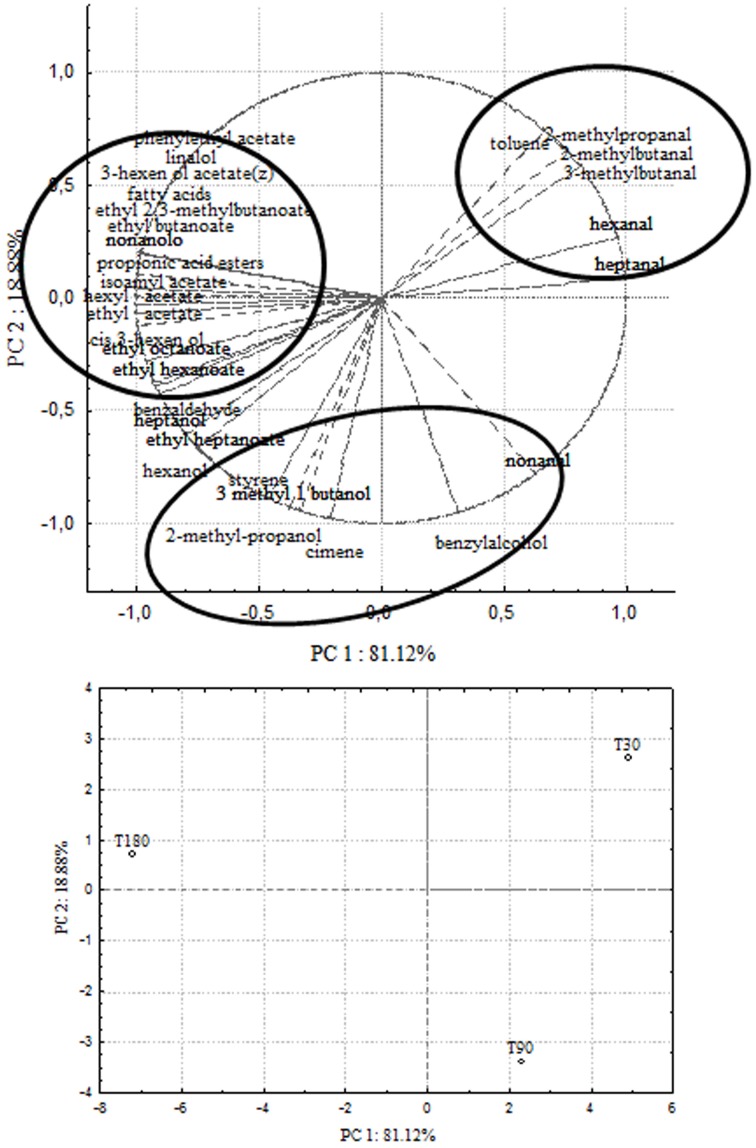
**PCA of volatile compounds associated with Cellina di Nardò fermented table olives**. PCA variables were the data obtained from the analysis of concentration and presence of volatile compounds at three different fermentation times. The figure displays the sample scores and variable loadings in the planes formed by PC1–PC2.

**Figure 6:**
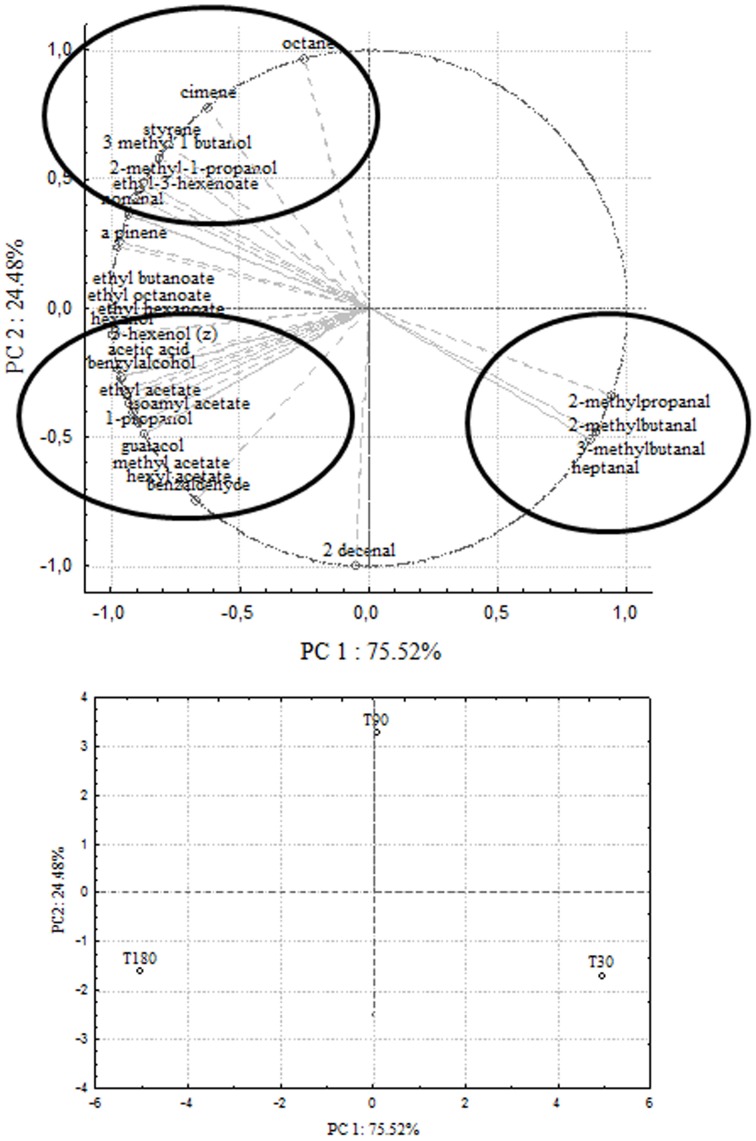
**PCA of volatile compounds associated with Leccino fermented table olives**. Score plot of variables (concentration of volatile molecules) and three different fermentation times in the plane formed by the first two principal components (PC1 against PC2).

In order to correlate chemical data with microbial population dynamics and to identify particular compounds to be suitable for monitoring olive fermentation, a PCA analysis was performed on the complete SPME/GC-MS data matrix of each olive sample. The analysis was carried out by plotting the mean values of each volatile compound (variables) at 3 different fermentation times (30–90–180 days of brining). Two bi-plots displaying PC1 vs. PC2 are illustrated in Figures 5, 6, which show the projection of the variables on the plane defined by the first and second principal components. In Cellina di Nardò and Leccino, the first principal component (PC1) explained 81.12 and 75.52%, respectively, of the total variability, between volatile compounds produced during fermentation and the second principal component (PC2) accounted for an additional 18.88% (Cellina di Nardò) and 24.48% (Leccino). The two planes made using the first two PCs (Figures 5, 6) indicated that the molecules were divided into three groups. In both table olive cultivars, one group, consisting of aldehydes, resulted closely related to the first stage of fermentation (T30); the second one consisted of higher alcohols (2-methyl-1-propanol; 3-methyl-1-butanol), styrene, and o-cymene associated with the middle stage of fermentation (T90) and the third one contained acetate esters linked to the final step in olive fermentation (T180).

## Discussion

Olive fermentation is a complex process, where the enzymes from olives interact with the metabolic activities of microorganisms through various biochemical pathways (McFeeters, 2004). The volatile compounds are produced in the brine by fermentation processes operated by microorganisms and in the fruit matrix by the action of either endogenous and microbial enzymes (produced by yeasts, lactic acid bacteria, etc.) (Sabatini et al., 2009). Volatile compounds in table olives belong to very heterogeneous group including higher alcohols, aldehydes, ketones, esters, fatty acids, monoterpenes, and hydrocarbons (Malheiro et al., 2011). However, limited data are present in literature on the compounds responsible for the organoleptic properties of table olives.

In the present paper, we have produced the molecular and technological characterization of LAB and yeast populations in Cellina di Nardò and Leccino spontaneous fermentations, as a first step for the production of autochthonous fermentation starters. Moreover, the evolution of the main physic- chemical parameters and volatile compounds during fermentations were studied in order to determine chemical descriptors, correlated with microbiological activities, that may be suitable for monitoring the outcome of the fermentation.

The microbiota of processed olives or brines include members of *Enterobacteriaceae, Clostridium, Pseudomonas, Staphylococcus*, LAB, yeasts, and occasionally molds (Heperkan, 2013). In agreement with data previously reported by Alves et al. (2012), our results indicated that, during the fermentations of both Cellina di Nardò and Leccino olives, some *Enterobacteriaceae* were initially present but after few days they resulted undetectable and were not detected at the end of the process. No *Clostridium* and *Pseudomonas* were revealed, probably because they were unable to survive till the end of the process due to the low pH values (<4.3, the maximum pH value established by the IOOC for preserving olives physicochemical characteristics (Montano et al., 2010). By contrast, yeasts were detectable throughout the fermentation process and finally, in the last month, the hitherto undetectable LAB appeared and carried out lactic fermentation. According to Arroyo-López et al. (2012a) and Bevilacqua et al. (2012), yeasts play a substantial role in the production of both green-treated olives and black naturally-fermented olives. Also, during Leccino and Cellina di Nardò olive fermentations, they could exert a fundamental role. Yeasts increased their concentration throughout both fermentation processes from 3.0 log CFU/ml (at the beginning) to 4.0–5.0 log CFU/ml (at the end). Since several studies have demonstrated that the action of indigenous yeasts during olive production process is able to enhance the quality of the final product (Aponte et al., 2012; Bevilacqua et al., 2012, 2013), a pre-selection and technological characterization of yeast populations associated with Cellina di Nardò and Leccino olives was carried out. Yeasts and LAB associated to Cellina di Nardò and Leccino fermented table olives were isolated and characterized. In a first step, isolated microorganisms were assayed in a “model brine,” formulated *ad hoc* for yeasts to replicate the industrial conditions of brine fermentation. To this purpose, starting from previous indications by Servili et al. (2006), an improved synthetic mixture of glucose, sodium chloride, and the mono-cyclic and poly-cyclic aromatic acids was used to grow yeast and LAB in low pH and temperature conditions. Then, the microbial isolates able to grow in model brine, were assayed for their the capacity to secrete beta-glucosidase (thus being able to rapidly eliminate oleuropein) and the inability to produce biogenic amines. Indeed beta-glucosidase contribute to olives debittering by degrading polyphenols such as oleuropein, and it was considered an interesting positive technological trait (Bevilacqua et al., 2012, 2013). Isolates that showed the above described abilities were evaluated also for the presence of lipase activity, that could improve the aromatic profile of fermented olives by increasing their free fatty acid content (Rodríguez-Gómez et al., 2012), and the absence of proteolytic activity, which could have a negative impact on olive quality because it is related to olive softening (Arroyo-López et al., 2012a). The third step in the proposed procedure consisted of the identification of the selected strains by sequencing a significant portion of their rRNA genes. In agreement with previous studies in Cellina di Nardò fermentations we identified *D. hansenii* (Nisiotou et al., 2010), *W. anomalus* (Nisiotou et al., 2010), *P. membranifaciens* (Coton et al., 2006; Bautista-Gallego et al., 2011) *D. carsonii* and *C. tartarivorans*, whereas in Leccino fermentations were detected *S. cerevisiae, P. membranifaciens, D. etchellsii, C. boidinii* and *Z. mrakii* (Arroyo López et al., 2006; Nisiotou et al., 2010).

Lactic acid bacteria are the most important group of bacteria in olives. They are able to convert fermentable sugars to lactic acid and other organic acids depending on their metabolic pathways. In fermented olives, homo fermentative LAB such as *Lactobacillus, Streptococcus*, and *Pediococcus* and hetero fermentative LAB such as *Leuconostoc* and some members of *Lactobacillus* (Abriouel et al., 2012; Randazzo et al., 2012) can be found. In particular, *Lactobacillus* spp. play a major role, while *Leuconostoc* and *Pediococcus* are involved to a lesser degree (Abriouel et al., 2011; Corsetti et al., 2012). In accordance with the above previous studies, bacterial isolates obtained from both Leccino and Cellina di Nardò fermentation belonged to *L. plantarum* species with a very limited number of *Kocuria* and *S. salitolerans* found in the second one. However, because the demonstrated beneficial effects for human health deriving by the application of microbial starters in table olive processing (Silva et al., 2011; Argyri et al., 2013; Bautista-Gallego et al., 2013; Blana et al., 2014), the probiotic activity of the characterized yeasts and LAB will be further investigated. Moreover, another trait that will be examined is the ability of the identified isolates to produce bacteriocins, since this feature can increase the competitiveness of the producer strain in food and contribute to prevent food spoilage obtaining longer shelf life and safety of the products reducing the use of chemical preservatives (Holzapfel, 2002; Leroy and DeVuyst, 2004; Arroyo-López et al., 2005).

The differences observed in microbial population in the two fermentations could be related to the different physico-chemical composition of the two olive cultivars. Preliminary data obtained in our laboratory indicated that Cellina di Nardò and Leccino olives differed in their chemical profile because Leccino is richer of organic acids (citric, tartaric, malic, succinic, acetic acid) and sugars (glucose, fructose, and sucrose) than Cellina di Nardò. Moreover, significant differences were detected also in volatile profiles of the two olive varieties: Leccino showed a 1.5-fold higher content of aldehydes than Cellina di Nardò and the presence of a high level of hydrocarbons, whereas Cellina di Nardò olives revealed a 2-fold and 3-fold higher contents of alcohols and terpens than Leccino, respectively (data not shown).

The results of this study showed that yeasts are responsible of the first part of fermentation (at least 90 days) and then LAB, together with yeasts, complete the process for the period ranging from 90th day to 180th day. Although different yeasts and bacteria species are responsible of the process, these data confirmed the microbial evolution described in spontaneous fermentation of Conservolea and Kalamàta olives (Bleve et al., 2015).

In order (i) to identify specific molecules suitable to be proposed as tools for process monitoring among the chemical compounds produced during fermentation and (ii) to correlate the results of physico-chemical analyses with the microbial metabolism, we optimized a novel procedure for the analysis of table olive and brine chemical composition. In particular, we used the solid-phase micro extraction (SPME), a technique widely used for the analysis of olive oil (Kanavouras et al., 2005; Temime et al., 2006; Baccouri et al., 2008) and table olive aroma compounds (Kalua et al., 2007; Aponte et al., 2010; Malheiro et al., 2011). The statistical analysis (PCA) of the outcome of the chemical assays allowed us to establish a correlation among the profiles of volatile molecules and microbial growth also giving indications of the molecules that can be considered helpful descriptors of each fermentation stage. Soluble sugars, such as glucose and fructose, are substrates for microbial fermentation giving primary and secondary metabolites responsible for the good organoleptic characteristics and the distinctive flavor of the final product. The prevalence of residual glucose over fructose could be related, besides varietal differences, to the fructophilic character of some non-*Saccharomyces* yeasts commonly found in brine. Independently of the total number of LAB, the great variability of lactic acid content could be related to many factors, such as the content of fermentable sugars, as well as the balance among species/strains present and their acidifying activity.

Analogously to the data observed in Conservolea and Kalamàta table olives, the first stage (30 days) of fermentation is mainly characterized by a high concentration of aldehydes (2+3-methylbutanal, 2-methylpropanal), that are very important compounds in fruits and vegetables and can contribute to characteristic herbaceous flavors. Italian cultivars presented lower concentrations of C2-, C4-, and C6-aldehydes and ketons than the two Greek varieties (Bleve et al., 2015).

The second fermentation period (90 days) was mainly characterized by the presence of higher alcohols, styrene, and terpenes. According to data reported in Tables 3, 4, olives and brines, during the fermentation process, showed changes in their sensory profile, with a decrease in the number of aldehydes and the appearance of new compounds. Indeed, aldehydes undergo a series of enzymatic transformations mediated by microbial isomerases and alcohol dehydrogenases, leading to C6 alcohols (Cavalli et al., 2004). The increase in isoamyl alcohols during the first fermentation step indicates the role of yeasts in driving this part of the process and suggests that these molecules are important markers of yeast metabolism. In particular, among alcohols, 2+3 methyl-1-butanol (isoamyl alcohol, fruity-winey notes), hexanol (fruity-green notes) and cis 3-hexen-1-ol (green notes) prevailed both in olives and brines. Higher alcohols (1-propanol and 2-methyl-1 propanol) derive from the reduction process of aldehydes, but can also be linked to the microbial deamination process of amino acids (McFeeters, 2004). In the two Italian table olive cultivars Cellina di Nardò and Leccino there was the absence of sesquiterpenes (farnesene and cubebene) and of monoterpene (cimene) found in the two Greek varieties Conservolea and Kalamàta. Moreover, Greek cultivars were richer of alcohols than Italian ones (Bleve et al., 2015).

The third fermentation stage (180 days) was characterized by a significant increase in alcohols/esters/fatty acids/hydrocarbons, probably due to the different pathway undertaken by enzymes produced by LAB, yeasts and/or other microorganisms. There was an increase in acetic acid, which is indicative of a heterolactic fermentation process to the microbial action, mainly operated by LAB. A considerable presence of acetate esters, acetic acid, propanoic, and 2-methylpropanoic acid negatively correlated to the PC1 semi-axis in Cellina di Nardò (80.44% variance) and Leccino (77.20% variance) olive maps, characterizing the sixth month of the process (T180) linked to the appearance of bacteria. Acetic acid is produced by yeast activity, but its concentration increased due to the activity of bacteria in the last stage of fermentation. Although the presence of short chain fatty acids is usually related to the appearance of negative odors, they can be very important for aromatic equilibrium because they are opposed to the hydrolysis of the corresponding esters, as already reported in wines (Bertrand, 1981; Edwards et al., 1990). Moreover, Greek cultivars were richer of esters than Italian ones, and the comparison of chemical profiles revealed the absence of ethyl lactate and the presence of 3-hexenol-acetate in Italian table olives (Bleve et al., 2015). Compounds identified as chemical descriptors are common not only for the two fermentations of Cellina di Nardò ad Leccino, but also for the two Greek cultivars of table olives (Bleve et al., 2015) and are strongly related to the activity and evolution of microorganisms (yeasts and LAB) during the process. Several authors reported the accumulation of hydroxytyrosol in the brine, as the main simple phenolic compound in olives at the end of fermentation (Romero et al., 2004; Ben Othman et al., 2008; Pistarino et al., 2013). Accumulation of hydroxytyrosol in the brine can be attributed to the ability of microorganisms to hydrolyze complex compounds such as oleuropein, that decreased during the process, by means of beta-glucosidase (Servili et al., 2006). Indeed in our samples, oleuropein was completely absent after the 30th day in Cellina di Nardò and after 90 days of fermentation in Leccino brines. According to the here reported results, yeasts play an important role in the olive debittering process. The different physico-chemical characteristics of each olive variety can be responsible of the differences observed at the end of fermentation in Italian and Greek cultivars. In fact, the Italian olives showed a richer profile of phenolic compounds than Greek olives (Bleve et al., 2015).

In conclusion, the chemical descriptors identified in this investigation represent the first evidence that specific molecules can be suggested as predictors for monitoring the fermentation process of black table olives produced by Greek method. For the first time, it has been described the technological and molecular characterization of yeast and LAB isolates associated to Cellina di Nardò and Leccino and it represents the first stage of a selection procedure for the production of mixed autochthonous fermentation starters. After assessment of the enzymatic traits and their ability to grow in model brines, two starter formulations, composed of *P. anomala*/*L. plantarum* (Cellina di Nardò) and *S. cerevisiae/L. plantarum* (Leccino) strains are going to be validated through olive industrial-scale fermentation.

Further work is now forthcoming in order to identify organoleptic descriptors and to set up a panel test for the evaluation of the Cellina di Nardò and Leccino olives.

## Author contributions

Substantial contributions to the conception and design of the work, acquisition, analysis, and interpretation of data (Gianluca Bleve, Francesco Grieco, Giovanni Mita, Maria Tasioula-Margari, Antonio F. Logrieco); acquisition, analysis and interpretation of data (Maria Tufariello, Miriana Durante, Ezio Perbellini, Francesca A. Ramires, Maria S. Cappello, Stefania De Domenico); drafting the work and revising it critically for intellectual content (Gianluca Bleve, Francesco Grieco, Giovanni Mita, Maria Tasioula-Margari). All authors approved the final version of the manuscript to be submitted for publication and agreed to be accountable for all aspects of the work in ensuring that questions related to the accuracy and integrity of any part of the work are appropriately investigated and resolved.

### Conflict of interest statement

The authors declare that the research was conducted in the absence of any commercial or financial relationships that could be construed as a potential conflict of interest.
